# Supplementary figures and images for: Potent antibody-dependent cellular cytotoxicity of a V2-specific antibody is not sufficient for protection of macaques against SIV challenge
Source: PLoS Pathog. 2024 Jan 22;20(1):e1011819. doi: 10.1371/journal.ppat.1011819 (PMC10833561; doi:10.1371/journal.ppat.1011819)

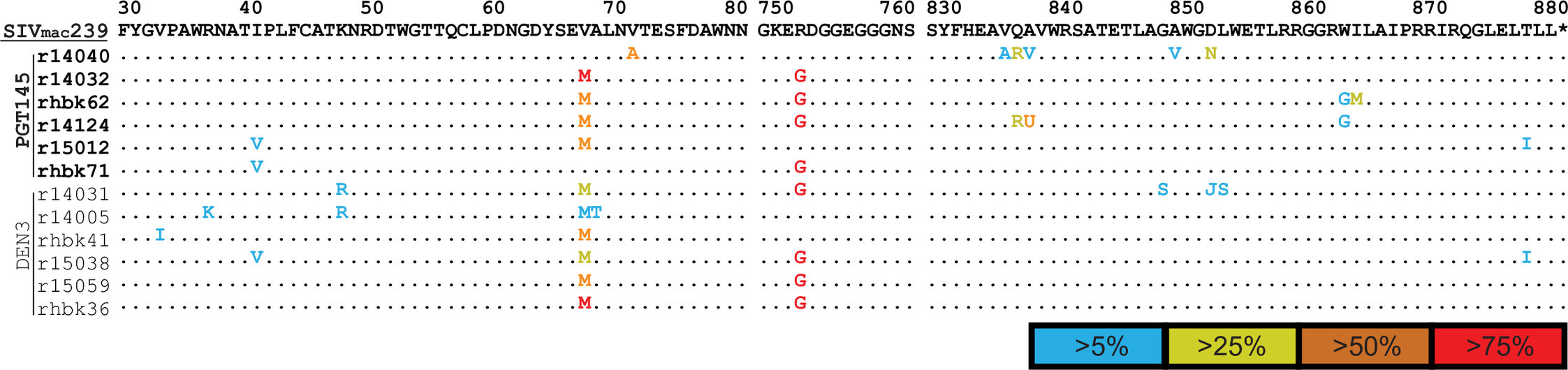

Supplement: S1 Fig — Viral RNA was isolated from plasma at week 3 post-infection and sequenced. The predicted amino acid sequences in Env from PGT145-treated animals (top, bold) and from DEN3-treated animals (bottom, plain text) were aligned to SIVmac239 Env. Regions of Env with substitutions in multiple animals are shown. Positions of identity are indicated by periods and amino acid differences are identified by their single letter code. Amino acid ambiguities are indicated with non-standard letters as follows: U = D/V, J = N/V. The frequencies of each substitution within the virus population are indicated by color. (TIF) [file ppat.1011819.s001.tif]

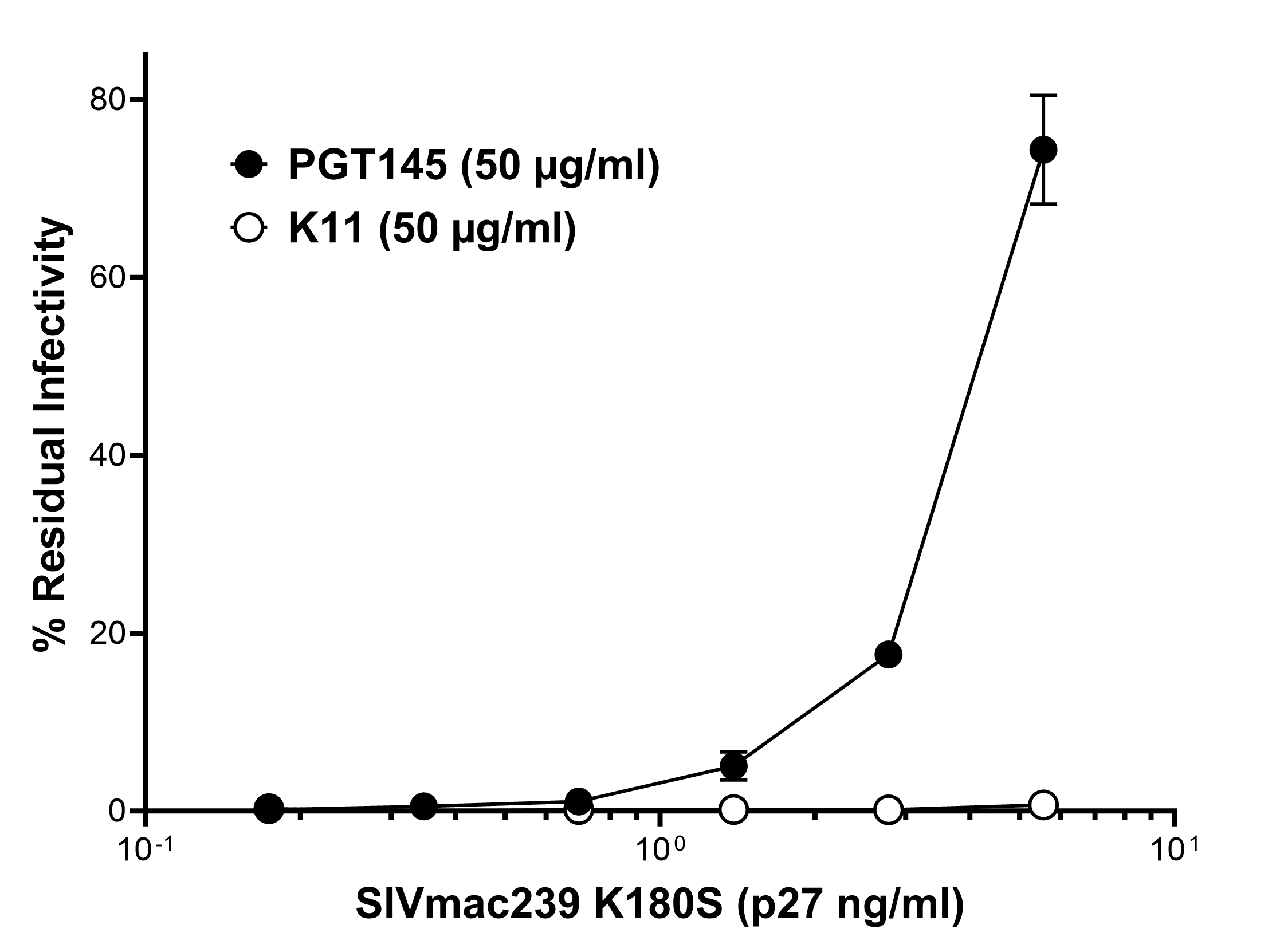

Supplement: S2 Fig — Two-fold dilutions of the SIVmac239 K180S challenge virus (starting at 5.56 ng/ml p27) were incubated in the presence of a constant amount of PGT145 and K11 (50 μg/ml) for one hour before the addition of TZM-bl cells. Luciferase activity in the TZM-bl cells as an indicator of SIV infectivity was measured after a 3-day incubation. The percentage of residual infectivity was calculated from the luciferase activity in presence of each antibody relative to maximal luciferase activity in the absence of antibody after subtracting background luciferase in uninfected cells. (TIF) [file ppat.1011819.s002.tif]

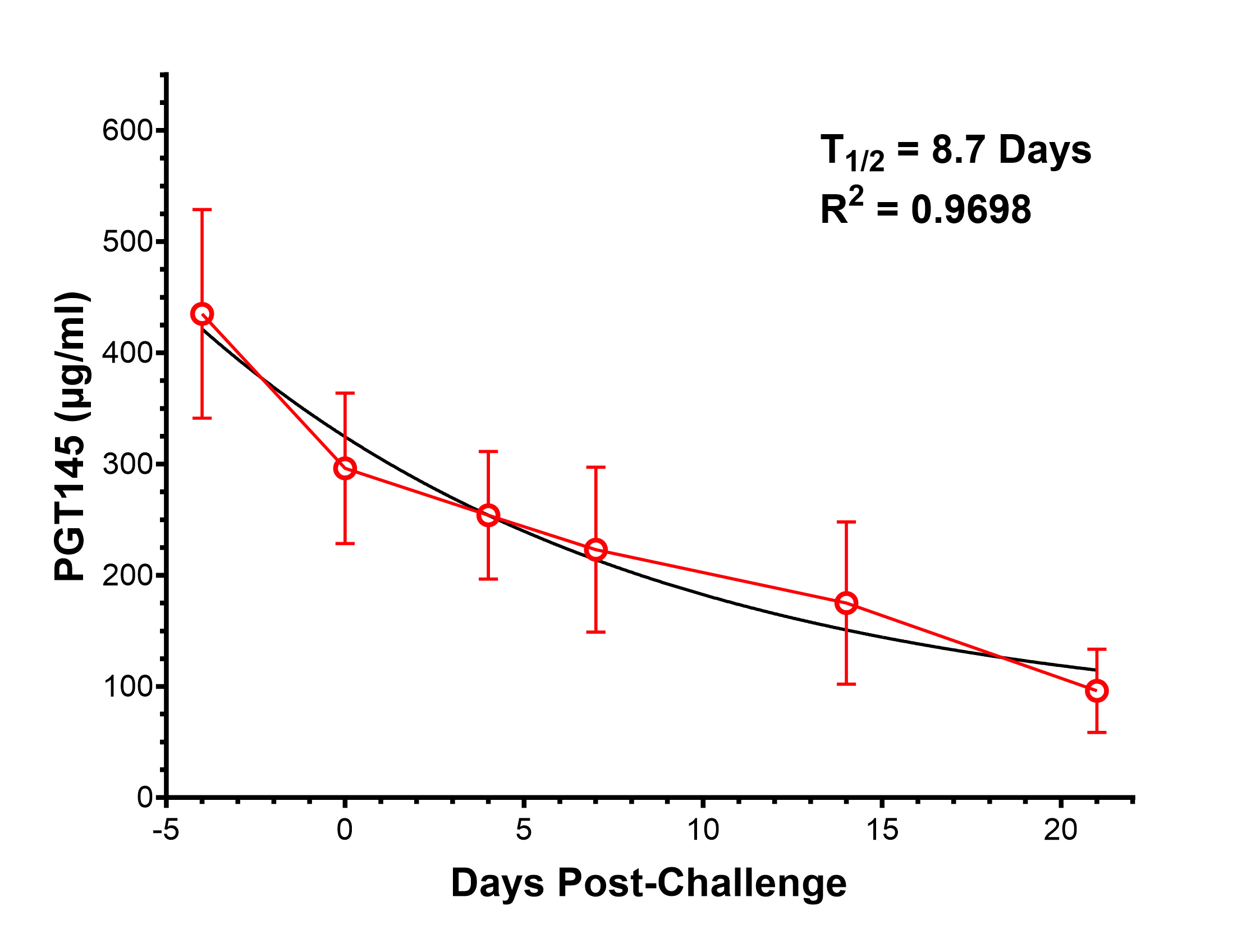

Supplement: S3 Fig — Serum concentrations of PGT145 in animals challenged with SIVmac239 K180S were measured by ELISA on plates coated with an anti-His antibody and captured 6-His-tagged HIV-1 BG505 SOSIP trimers. Error bars indicate standard deviation of the mean. The data was analyzed by nonlinear regression. The best-fit cure is shown in black with an R2-value of 0.9698 and an estimate antibody half-life of 8.7 days. (TIF) [file ppat.1011819.s003.tif]
